# Clinical effects of an optimised care program with telehealth in heart failure patients in a community hospital in the Netherlands

**DOI:** 10.1007/s12471-015-0692-7

**Published:** 2015-05-07

**Authors:** W. Veenstra, J. op den Buijs, S. Pauws, M. Westerterp, M. Nagelsmit

**Affiliations:** 1 Department of Cardiology, Scheper Hospital Emmen, Boermarkeweg 60, 7824 AA Emmen, The Netherlands; 2 Philips Research Europe, High Tech Campus 34, 5656 AE Eindhoven, The Netherlands; 3 Philips Healthcare Benelux, Boschdijk 525, 5621 JG Eindhoven, The Netherlands

**Keywords:** Care coordination, Heart failure, Telehealth

## Abstract

**Background:**

Our hypothesis was that telehealth in combination with an optimised care program coordinated amongst care professionals in primary, secondary and tertiary care can achieve beneficial outcomes in heart failure. The objective was to evaluate the clinical effects of introduction of telehealth in an optimised care program in a community hospital in the north of the Netherlands.

**Methods:**

We compared the number of unplanned admissions for heart failure in the year before and after adding telehealth to the optimised care program. Furthermore, blood pressure and N-terminal pro-B-type natriuretic peptide (NT-proBNP) levels were evaluated at baseline and 3, 6 and 12 months after telehealth. Quality of life and knowledge about the disease were regularly evaluated via surveys on the telehealth system.

**Findings:**

The number of unplanned admissions for heart failure decreased from on average 1.29 to 0.31 admissions per year after telehealth introduction. Blood pressure decreased independent of medication and NT-proBNP levels improved as well. Quality of life increased during the telehealth intervention and disease knowledge remained high throughout the follow-up period. Unplanned admissions that remained after telehealth introduction could be accurately predicted at baseline by a multivariate regression model.

## Introduction

Congestive heart failure (CHF) is a complex clinical syndrome, which represents an increasingly common health problem for patients, healthcare providers and reimbursing agencies. The continuing ageing of the population is expected to increase the prevalence of heart failure in the Netherlands from 120,000 (~ 1 % of the population) in 2008 to approximately 200,000 in the coming decade [1]. In 2011, the number of hospital admissions with heart failure as the primary discharge diagnosis was almost 30,000 [1]. Of all patients who were admitted once for heart failure, 14 % of them were re-admitted to the hospital within 6 months after their first index admission [2].

Guidelines of the European Society of Cardiology (ESC) recommend a multidisciplinary approach that coordinates care along the continuum of CHF, often implemented as in-person follow-up visits [3]. Recently, alternative approaches such as telehealth and remote monitoring via cardiac implanted electronic devices [4, 5] have been proposed to assess physiological parameters related to CHF exacerbation more frequently, thereby enabling remote disease management [6]. Telehealth uses various information and communication technologies to assist in the management of an existing long-term medical condition of a patient at home by delivering clinical care and non-clinical services, including education on the disease and the capture and transmission of questionnaire data and vital signs. However, the optimal approach to non-invasive remote monitoring is uncertain, and an ESC guideline recommendation is not yet supported because the randomised controlled trials (RCTs) performed to date have given inconsistent results [3].

Telehealth for heart failure has had mixed and heterogeneous effects across studies, necessitating further evaluation within specific healthcare systems and patient populations. A systematic review on telehealth efficacy in RCTs concluded that telehealth reduced all-cause mortality by 34 % and CHF-related hospitalisations by 21 % and improvements in secondary endpoints such as quality of life and disease knowledge were also observed [7]. However, some studies included in the systematic review did not corroborate the beneficial effects of telehealth. The TEN-HMS study, the world’s first multi-centre RCT for telemonitoring in the UK, Germany and the Netherlands, showed that the use of telemonitoring reduced all-cause mortality by 29 % in a 240-day follow-up, though this was not significant when comparing with usual care [8]. In the HartMotief study, which was a multi-centre trial in eight Dutch hospitals, telemonitoring also failed to reduce mortality in a 288-day mean follow-up [9]. Furthermore, some later studies were not able to demonstrate telehealth efficacy. In the TIM-HF study, telehealth was implemented by means of a call centre and compared with an already excellent conventional care. In this study, telehealth failed to reduce all-cause mortality or a combined endpoint of cardiovascular mortality or hospitalisation in a mean follow-up of 21 months [10]. The Tele-HF study was unable to show reduction in mortality and admission for a structured telephone support system in a 6-month follow-up [11]. Poor patient compliance may have had an impact on the findings, as the use of the system was laborious for end users. The Whole System Demonstrator (WSD), a large RCT of telehealth involving 3230 patients with CHF, chronic obstructive pulmonary disease or diabetes mellitus in the UK, showed that telemonitoring lowered mortality and emergency admissions by 45 and 20 %, respectively, in comparison with usual care in a 1-year follow-up [12]. Cost-effectiveness analysis of WSD results did not show telehealth to be a cost-effective alternative to usual care [13], but unfortunately the decrease in mortality in the telehealth group was not taken into account in this analysis. Even though most available data suggest that telehealth is a promising strategy for improving disease management of CHF patients, more data are needed to determine the most advantageous approaches.

The first objective of this study was to assess the impact of telehealth-based disease management on unplanned admissions, disease severity and quality of life in CHF patients. The unique aspect of this study was its setting: a single community hospital in the north of the Netherlands, where cardiologists and nurse practitioners collaborate within the hospital outpatient heart failure unit. Furthermore, primary care, local home care agencies and nursing homes play a key role in the care protocol of CHF patients through their general practitioners, home care nurses and physicians. The second objective was to determine, which patient characteristics were predictive of unplanned admissions in a telehealth program. As unplanned readmissions are costly, a predictive score to quantify the risk of readmission would help clinicians to identify patients who might benefit from more intensive post-discharge care, even beyond telemonitoring.

## Materials and methods

### Study design

In this prospective study, 102 CHF patients were enrolled in an optimised care program with telehealth between 3 April 2011 and 10 May 2013. A pre-post study design was used to evaluate the effect of the telehealth intervention without the use of a control group. The primary outcome, number of unplanned hospitalisations for heart failure, was measured 1 year before and 1 year after introduction of the program. Unplanned admissions were considered admissions that could not be delayed beyond 24 h because of symptoms ascribed to heart failure by the attending physician or nurse practitioner. Secondary outcomes, blood pressure and NT-proBNP levels, were measured at enrolment and after 3, 6 and 12 months. Blood pressure was measured using an automatic upper arm cuff (A&D Medical, San Jose, CA, USA). NT-proBNP analysis was performed with an immunoassay (Elecsys proBNP, Roche Diagnostics, Almere, the Netherlands).

Patients discharged after an admission with the primary diagnosis of heart failure or outpatients after an episode of new or worsening heart failure, as judged by the attending cardiologist because of clinical symptoms and NT-proBNP levels elevated above normal limits, were recruited to participate in the study. Patients who were in New York Heart Association (NYHA) class II–IV, were in a stable condition, had good command of the Dutch language and were in possession of a television set were eligible for inclusion. Patients who were unable to provide informed consent were excluded from the study. Out of 105 patients who were asked to participate in the study, 102 (97 %) gave their informed consent.

The study was conducted in a CHF outpatient unit of a local community hospital in the north of the Netherlands (Scheper Hospital, Emmen, the Netherlands). The unit’s cardiologists and nurse practitioners provide care to heart failure patients in the region, involving the patient’s general practitioner, nurses from a local home care agency and physicians at the nursing home, if patients reside at such facilities. This structured service was already in place before patients were enrolled in the telehealth program. A detailed schematic diagram of the optimised care pathway including telehealth can be found elsewhere [14].

Staff had prescription and treatment authorisation, preventing delay in response-treatment times and preventing burden on the workload of the cardiologist. The telemonitoring system was used to obtain and check patient vital sign measurements (blood pressure, pulse, weight) on a daily basis. All members of the cardiology department had access to the telemonitoring system, allowing them to check vital sign measurements at every moment and react accordingly.

Retrospective data of 1 year on CHF-related admissions were gathered as well as demographics, clinical status and treatment and physical examination data. All patients were followed up for a period of 1 year with additional visits as required in case of deterioration. Follow-up visits at the outpatient unit took place 3, 6 and 12 months (study end) after enrolment. The nurse practitioners and cardiologists participating in the study were the same as those involved in the patients’ everyday care before the study. During the study period, all patients continued to receive the standard care provided to patients at the CHF outpatient unit. Patients received primary care from their own general practitioner.

The study was approved by the hospital ethics committee. All participants gave informed written consent.

### Telehealth intervention

The Motiva telehealth system (Philips Healthcare, Best, the Netherlands) was used and included a secured broadband home TV channel providing educational material, reminders of medication, health-related surveys and motivational messages to encourage the prescribed lifestyle regimen. Patients were given automated devices for daily measurements of blood pressure, heart rate and weight at home. A nurse practitioner evaluated the measurements every day using a dedicated clinical user interface. With tailored alarm settings, the nurse practitioner could identify which patients exceeded the alarm limits and needed extra attention. The situation was evaluated with a phone call or extra visit and if necessary the nurse practitioner altered the treatment.

Patients also received scheduled educational videos covering topics such as symptoms, daily care, aetiology, medication, compliance, relapse prevention, cholesterol management, hypertension, lifestyle, nutrition, vaccinations and alcohol consumption. Questionnaires on quality of life (Minnesota Living with Heart Failure, MLHF) and disease knowledge (Dutch Heart Failure Knowledge Survey, DHFKS) were sent every 8 and 12 weeks, respectively. MLHF measures the effects of symptoms, functional limitations and psychological distress on an individual’s quality of life due to heart failure and its treatment [15]. DHFKS covers items concerning heart failure knowledge in general, knowledge on heart failure treatment and symptoms [16]. Based on the answers, the nurse practitioner could decide to send the patient a video he/she believed could be valuable for the patient to review.

### Statistical analysis

The number of unplanned admissions for heart failure in the period of 12 months before telemonitoring (the index admissions were not included in the counts) was compared with the number of admissions in the period of 12 months after telemonitoring was introduced with a rate ratio test [17]. The incidence rate ratio is the rate of unplanned admissions in the pre-phase divided by the rate of unplanned admissions in the post-phase. The rate ratio test tests the null-hypothesis that the incidence rate ratio is equal to 1.0.

Repeated measures analysis of variance (ANOVA) followed by Tukey’s post-hoc comparison of means using α = 0.05 was used to determine significant deviations in blood pressure, NT-proBNP levels, knowledge level and quality of life from baseline. The method by Cousineau [18] was used to derive 95 % confidence intervals (CIs) for visualisation in charts. Repeated measures ANOVA was carried out to test for the effect of medication use and time on the telehealth program on blood pressure. The interaction between medication use and time on telehealth was included in this analysis to determine if the decrease in blood pressure over time was affected by medication.

It was further determined that patient characteristics at baseline could be used to provide a prediction of unplanned admission for heart failure within 12 months. A multivariate logistic regression model was developed on data from 80 patients chosen at random. The model variables were chosen using a forward selection approach, where the criterion used was to maximise the area under the receiver operator characteristic curve (AUC). Furthermore, to more accurately account for the non-linear relation between age and outcome, both age and age squared were included as predictors in the model. A cut-off level for NT-proBNP of 2500 pg/l was used to model the increased outcome in patients with NT-proBNP levels beyond 2500 pg/l. The model was evaluated using the AUC on the hold-out set of 22 patients.

## Results

### Baseline

Out of 105 heart failure patients considered eligible, 102 patients (97 %) gave informed consent. Patients were on average 75 ± 12 years and 60 % were female patients (Table 1). The majority of heart failure cases were a result of ischaemic heart disease. In this population, 74 patients (73 %) were in NYHA class III or IV and NT-proBNP values averaged 4433 pg/l. Implantable pacing devices were present in 36 % of the patients; 89 % of patients had valvular disease and nearly half of patients had atrial fibrillation. Medication treatment at baseline covered most patients and can be considered excellent with 80 % of the patients on beta-blockers, 94 % on diuretics and 78 % on angiotensin-converting enzyme (ACE) inhibitors.

**Table 1:** Baseline characteristics of the 102 heart failure patients

Age	75 ± 12 Years (47–91)
*Gender*	
Female	61 (60 %)
Male	41 (40 %)
*Heart failure aetiology*	
Ischaemic	62 (61 %)
Non-ischaemic	40 (39 %)
*NYHA class*	
II	28 (27 %)
III	57 (56 %)
IV	17 (17 %)
eGFR	49.0 ± 12.0 ml/min
*Blood pressure/heart rate*	
Systolic	128.3 ± 16.1 mmHg
Diastolic	77.6 ± 14.0 mmHg
Pulse	73.4 ± 15.3 bpm
N-terminal pro-B-type natriuretic peptide	4433 ± 5720 pg/l
*Medications*	
β-blockers	82 (80 %)
Diuretics	96 (94 %)
ACE inhibitors	80 (78 %)
Implantable devices	37 (36 %)
*Affected valves*	
Mitral	89 (87 %)
Aortic	16 (16 %)
Tricuspid	59 (58 %)
Atrial fibrillation	47 (46 %)

*NYHA* New York Heart Association, *eGFR* estimated glomerular filtration rate, *ACE* angiotensin-converting enzyme

### Clinical outcomes

There were 132 unplanned admissions for heart failure in the 12 months before telehealth enrolment, or an average of 1.29 admissions per patient in the pre-phase. In the 12 months after telehealth introduction, there were 32 unplanned heart failure admissions, or on average 0.31 per patient, i.e. a reduction of 76 % in unplanned heart failure admissions. The incidence rate ratio was found to be statistically significantly different from 1 (rate ratio: 4.1, 95 % CI: [2.8–6.3], *p* < 0.001). In the pre-phase, 87 patients (85 %) had one or more unplanned admissions for heart failure, while only 31 patients (30 %) had one or more admissions in the post-phase. The number of days spent in hospital for all-cause admissions decreased from 975 in the 12 months before telehealth enrolment to 662 in the 12 months after telehealth introduction.

Clinical effects of the telehealth program were determined by measuring systolic and diastolic blood pressure and NT-proBNP levels at baseline and after 3, 6 and 12 months in the program (Table 2). All 102 patients had 4 follow-ups, but systolic blood pressure was not recorded in 29 cases, diastolic blood pressure was not recorded in 31 cases and NT-proBNP level was not recorded in 55 cases. Systolic blood pressure decreased significantly by 7.9 mmHg (F(3,281) = 25.1, *p* < 0.001), while diastolic blood pressure decreased significantly by 5.0 mmHg (F(3,279) = 22.6, *p* < 0.001) after 12 months in the telehealth program as compared with baseline. For NT-proBNP level, a significant decrease over time was found from 4433 ± 5720 at baseline to 2877 ± 3128 pg/l at 12 months, a reduction of 35 % (F(3,250) = 10.9, *p* < 0.001).

**Table 2:** Population means ± standard deviation of systolic and diastolic blood pressure and N-terminal pro-B-type natriuretic peptide (NT-proBNP) values in 102 patients at baseline and during the telehealth program

	Baseline	3 Months	6 Months	12 Months
Systolic blood pressure (mmHg)	128.3 ± 16.1	124.5 ± 18.3*	121.4 ± 16.7*	120.4 ± 15.7*
Diastolic blood pressure (mmHg)	77.6 ± 14.0	74.2 ± 14.1*	73.1 ± 14.1*	72.6 ± 12.3*
NT-proBNP (pg/l)	4433 ± 5720	3875 ± 5015*	3001 ± 3274*	2877 ± 3128*

*p < 0.05 based on repeated measures analysis of variance with Tukey’s post-hoc comparison of means of 3, 6 or 12 months vs. baseline

**Fig. 1:**
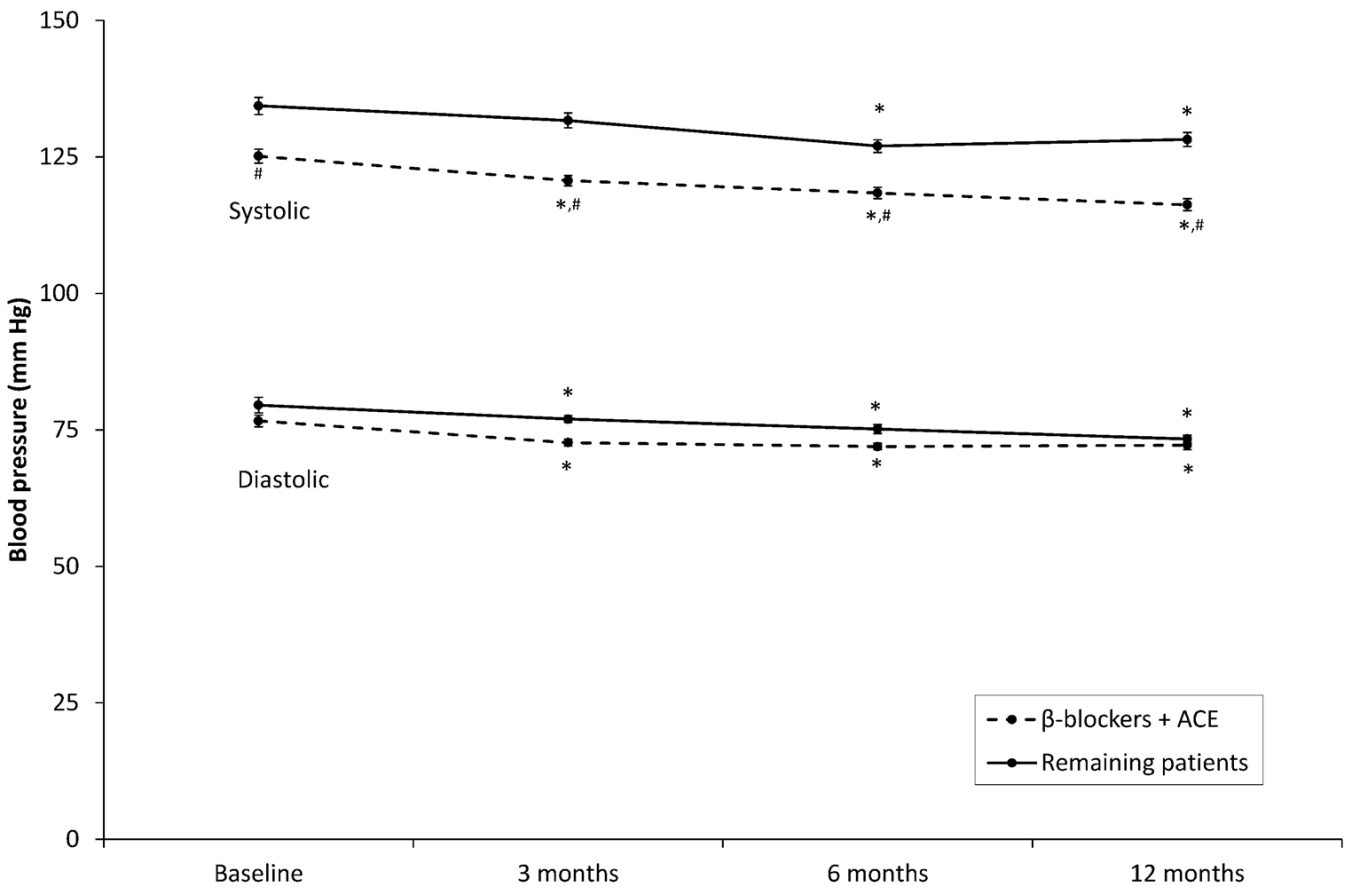
Systolic and diastolic blood pressure in patients who were prescribed both β-blockers and angiotensin-converting enzyme inhibitors compared with the remaining patients. *Error bars* represent within-subject 95 % confidence intervals. * statistically significantly different from baseline (*p* < 0.05). ^*#*^ statistically significantly different from patients without both medications

A total of 67 patients (66 %) were prescribed both β-blockers and ACE inhibitors. In patients using both medications, systolic blood pressure was significantly lower than in other patients (F(1,93) = 10.2, *p* < 0.05), but diastolic blood pressure was not (F(1,93) = 1.2, *p* = 0.28). Repeated measures ANOVA including the interaction between use of both medications and time on the telehealth program demonstrated that the combination of β-blockers with ACE inhibitors did not have a statistically significant effect on the decrease in systolic blood pressure (F(3,278) = 1.2, *p* = 0.31) or diastolic pressure (F(3,276) = 1.1, *p* = 0.33) over time (Fig. 1).

### Quality of life and disease knowledge

**Fig. 2:**
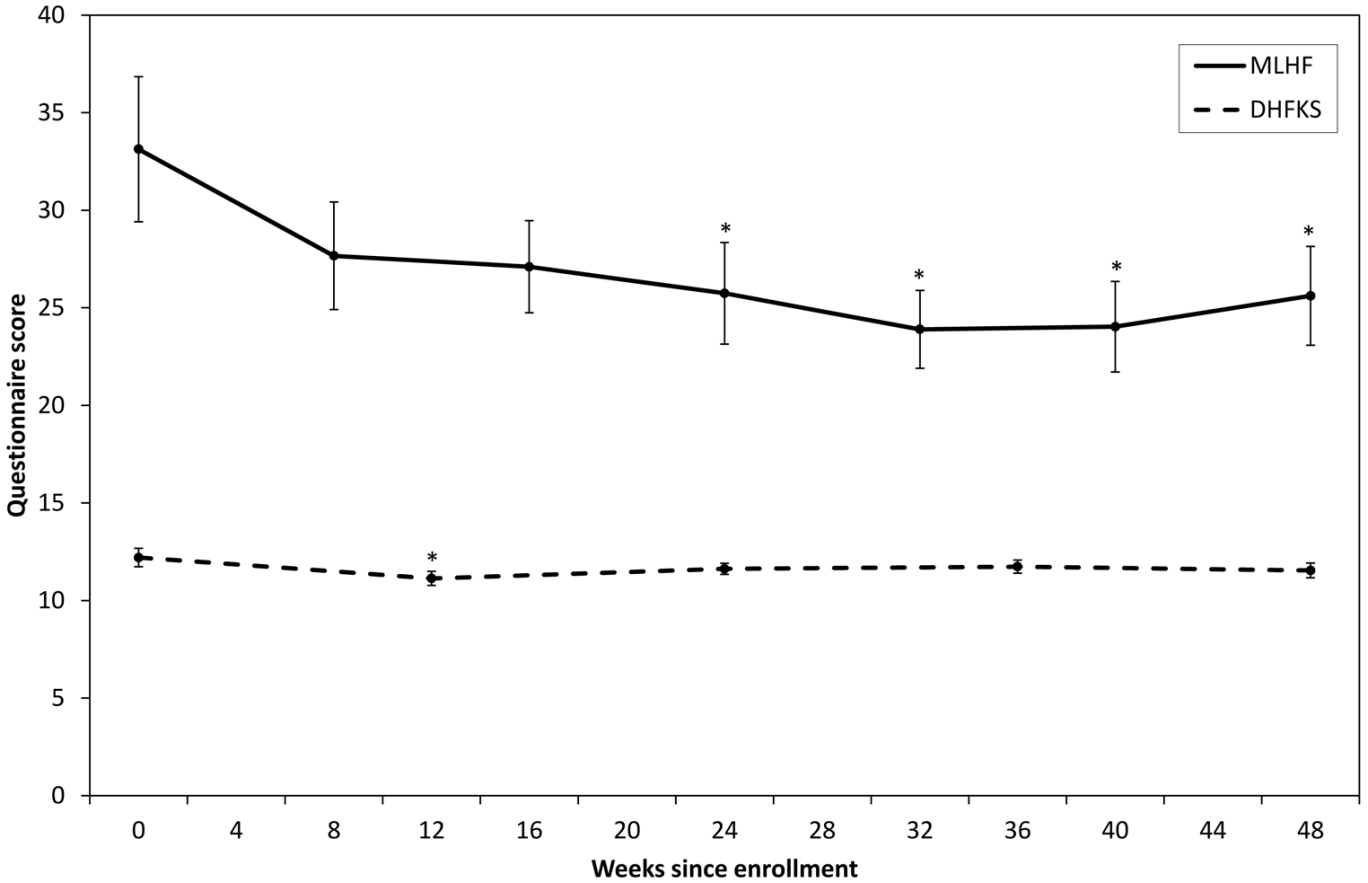
Mean responses to the Minnesota Living with Heart Failure (MLHF) and Dutch Heart Failure Knowledge Survey (DHFKS) questionnaires at different follow-up times. DHFKS scores range from 0 to 15 with a high score reflecting better disease knowledge. MLHF scores range from 0 to 105 with a low score reflecting a better health-related quality of life. *Error bars* represent within-subject 95 % confidence intervals. * statistically significantly different from baseline (*p* < 0.05)

Quality of life was assessed by the MLHF survey with complete observations in 86 patients; a higher MLHF score corresponds to a lower quality of life. Mean total MLHF scores decreased over time (Fig. 2), which was found to be statistically significant starting from 24 weeks after baseline (F(6,510) = 4.6, *p* < 0.001). Disease knowledge was assessed by the DHFKS survey with complete observations in 88 patients. The reported knowledge level was found to be consistently high across all follow-up times from 12.2 ± 0.5 at baseline to 11.5 ± 0.4 at 48 weeks (Fig. 2). The knowledge level at baseline was slightly but significantly higher than the knowledge level during the first follow-up (F(4,348) = 3.3, *p* < 0.05). There were no significant differences with baseline at the other follow-up times.

### Predictive analysis

A total of 31 patients (30 %) had one or more admissions for heart failure. Odds ratios for the multivariate logistic regression model are given in Table 3. AUC for the model development set was 0.90 and the AUC for the hold-out set was 0.93.

**Table 3:** Odds ratios of variables in the multivariable logistic regression model predicting unplanned admissions for heart failure in the next 12 months

Model variable	Odds ratio	95 % CI
Age	8.0*	2.8-23.1
Age^2^	0.98*	0.98-0.99
NYHA class IV	32.2*	1.4-722.8
N-terminal pro-B-type natriuretic peptide > 2500 pg/l	11.8*	1.5-89.9
Any implantable pacing device	0.10*	0.02-0.64
Heart failure admissions in past 12 months	8.3*	2.4-29.1

95 % confidence intervals (CI) are given

Model was developed using data from 80 randomly selected patients

*NYHA* New York Heart Association

*p < 0.05

## Discussion

In line with previous findings [19], the number of unplanned admissions for heart failure decreased in patients enrolled in a disease management program after telemonitoring was added to the program. Furthermore, blood pressure decreased independently of medication and NT-proBNP level decreased significantly, a trend that was observed earlier in internet-based home monitoring [20]. Finally, disease-specific knowledge was maintained at a high level and quality of life increased during the telehealth intervention.

Interventions using telehealth technology have the potential to reduce avoidable readmissions, and the evidence base for its beneficial effect is growing [12, 21]. However, various previous RCTs on the effect of telehealth showed disappointing results [10, 11, 22]. For instance, in the TEHAF study, the combined endpoint of heart failure admission and all-cause mortality was similar for both control and intervention groups. This could be in part attributed to already excellent care management in the control groups of these studies [23]. While multicentre RCTs are the gold standard of clinical evidence, a rigorous RCT is also very restrictive and telehealth results may be more superior when there is freedom in the implementation [24]. Therefore, reporting the results of this local telehealth project may contribute to the further development and critical evaluation of these systems in daily practice.

By providing education to promote change in patient behaviour for proper self-management, telehealth may have improved clinical indicators and quality of life [25]. Blood pressure readings improved over time in line with previous findings (see Paré et al. [26] for a review). We did not investigate if any medication adjustments triggered by telemonitoring affected blood pressure. Continuous surveillance of vital sign data in addition to usual care allowed for an immediate and adequate response to deviations from the accepted limits. As described previously [27], we found access to patient medical records for the staff who monitored the data to be a crucial factor in care coordination.

Predicting admissions in heart failure patients is difficult [28]; however, we showed that unplanned admissions in the next 12 months could be accurately predicted from baseline measurements for CHF patients who were in the telehealth program using multivariable logistic regression. Strong predictors included NYHA class IV and elevated NT-proBNP levels, which are indicative of increased disease severity. Emergency admissions may not be avoidable by a telehealth program alone in these patients.

Main limitation of the current study is the pre-post design without a control group. Therefore, one needs to be cautious when interpreting these results as being brought about by the telehealth program. The number of admissions on remeasurement is likely to be lower due to the statistical phenomenon of regression to the mean. An estimation of this effect [29] led us to believe that only 17 % of the observed reduction in unplanned admissions could have been explained by this effect. Furthermore, a learning effect may have occurred by sending the MLHF and DHFKS questionnaires every 8 and 12 weeks. In addition, based on the answers of the DHFKS, the nurse practitioner could decide to send the patient a relevant educational video. This may have influenced the DHFKS score as an outcome measure.

In the Netherlands, virtually all hospitals work with a CHF clinic staffed with either nurses or nurse practitioners, as it is a performance indicator monitored by the Dutch Healthcare Inspectorate [30]. Provided patient records are electronically available, the use of telemonitoring and implementation of its data in day-to-day care can be done without a substantial increase in workload for the nursing staff. However, contrary to nurse practitioners, nurses are not allowed to prescribe medication, which could impair a quick response.

In future, we are looking to further expand the current optimised care program with telehealth to other hospitals in the region. However, the inadequacy of reimbursing systems in the Netherlands to pay for this type of coordinated care constitutes a potential threat to its wider implementation, thereby possibly withholding improved chronic care from patients who could benefit and missing the opportunity to reduce overall costs of the care for CHF patients.

### Funding sources

None.

### Conflict of interest

Jorn op den Buijs, Steffen Pauws and Murk Westerterp are employed by Royal Philips.
